# Genome-Wide Spectra of Transcription Insertions and Deletions Reveal That Slippage Depends on RNA:DNA Hybrid Complementarity

**DOI:** 10.1128/mBio.01230-17

**Published:** 2017-08-29

**Authors:** Charles C. Traverse, Howard Ochman

**Affiliations:** Department of Integrative Biology, University of Texas, Austin, Texas, USA; The Sanger Institute

**Keywords:** *Buchnera*, elongation complex, error rates, *Escherichia coli*, mutation, RNA polymerase, transcription, transcription slippage

## Abstract

Advances in sequencing technologies have enabled direct quantification of genome-wide errors that occur during RNA transcription. These errors occur at rates that are orders of magnitude higher than rates during DNA replication, but due to technical difficulties such measurements have been limited to single-base substitutions and have not yet quantified the scope of transcription insertions and deletions. Previous reporter gene assay findings suggested that transcription indels are produced exclusively by elongation complex slippage at homopolymeric runs, so we enumerated indels across the protein-coding transcriptomes of *Escherichia coli* and *Buchnera aphidicola*, which differ widely in their genomic base compositions and incidence of repeat regions. As anticipated from prior assays, transcription insertions prevailed in homopolymeric runs of A and T; however, transcription deletions arose in much more complex sequences and were rarely associated with homopolymeric runs. By reconstructing the relocated positions of the elongation complex as inferred from the sequences inserted or deleted during transcription, we show that continuation of transcription after slippage hinges on the degree of nucleotide complementarity within the RNA:DNA hybrid at the new DNA template location.

## IMPORTANCE

The high level of mistakes generated during transcription can result in the accumulation of malfunctioning and misfolded proteins which can alter global gene regulation and in the expenditure of energy to degrade these nonfunctional proteins. The transcriptome-wide occurrence of base substitutions has been elucidated in bacteria, but information on transcription insertions and deletions—errors that potentially have more dire effects on protein function—is limited to reporter gene constructs. Here, we capture the transcriptome-wide spectrum of insertions and deletions in *Escherichia coli* and *Buchnera aphidicola* and show that they occur at rates approaching those of base substitutions. Knowledge of the full extent of sequences subject to transcription indels supports a new model of bacterial transcription slippage, one that relies on the number of complementary bases between the transcript and the DNA template to which it slipped.

## INTRODUCTION

In addition to the errors that occur during DNA synthesis, which form the basis for adaptation and heritable genetic variation, nonheritable errors are generated during the process of transcription. These transient errors are produced at rates that are orders of magnitude higher than replication error rates (1–4), such that a cell will invariably express a subset of transcripts that do not match the encoded sequence. This nonheritable variation is most often considered deleterious, since it can burden the cell with faulty or misfolded proteins in a manner similar to DNA mutations. Transcription errors can also result in collisions between replication and transcription machineries, thereby generating double-strand breaks in the chromosome and abortion of the transcript (5–8). It has also been proposed that transcription errors might somehow provide beneficial variation during times of stress (9–12). Nonetheless, because transcription errors are not stable across generations, study of their incidence and patterns of occurrence has traditionally been difficult.

Transcription error rates were originally measured with reporter genes engineered with premature stop codons, such that a specific transcription error would convert the sequence to produce a functional reporter protein (1, 13). Recently, a high-throughput sequencing approach expanded the spectrum of transcription errors from assaying a single site in a reporter gene to all protein-coding nucleotides in the transcriptome (14, 15). This technique, which relies on a unique library preparation method, allows direct quantification of transcription errors without contamination by the sequencing errors that typically befall transcriptome sequencing methodologies. Results from a study in which this method was applied revealed a transcription base substitution rate in *Escherichia coli* of ~8 × 10^−5^ per nucleotide that was relatively constant across different growth states and growth phases (4). Whereas this approach can provide accurate, genome-wide measurements of transcription errors, all sequencing-based studies in bacteria have been confined to the detection of base substitutions and have ignored transcription insertions and deletions (indels) (2–4, 16).

Transcription indels may be more detrimental than base substitutions, because individual nucleotide changes only alter a single amino acid and are often silent, whereas indels can involve multiple amino acids and usually disrupt the reading frame. The indels generated during transcription are generally thought to occur through the forward or backward slippage of the actively transcribing RNA polymerase (elongation complex) along the template DNA, causing a portion of the template to be either skipped (resulting in a deletion) or retranscribed (leading to an insertion) (17–20). Previous work exploited this slippage mechanism as a way of detecting transcription indels by engineering reporter genes with homopolymeric runs that, upon slippage, restored the proper reading frame (17–20). Although these studies yielded information about relative indel rates in certain homopolymeric tracts, such repeats are inherently error-prone and are not likely to represent the indel rate in coding sequences, which only rarely contain long homopolymeric runs (21).

The focus on error-prone repeats led to the notion that transcription indels occur primarily at homopolymeric runs (17–20), which are selectively removed from genomes (22). In the present study, we evaluated the occurrence of transcription indels throughout the genome, and we gained insights into the substrates and mechanism of transcriptional slippage. Because most information concerning transcription slippage has focused on homopolymeric runs, we compared the rates and patterns of indels in the transcriptomes of *E. coli*, which has an equitable occurrence of each nucleotide, and a low-G+C bacterial endosymbiont, *Buchnera aphidicola*, whose genome is greatly enriched in long tracts of adenosine and thymine. We found that while insertions predominated in homopolymeric runs in both species, deletions occurred in more complex sequences. These results led us to develop a general model of transcription slippage that is driven by RNA:DNA hybrid complementarity at the site of the new DNA template.

## RESULTS

### Rates of transcription-induced insertions and deletions across the transcriptome.

**FIG 1:**
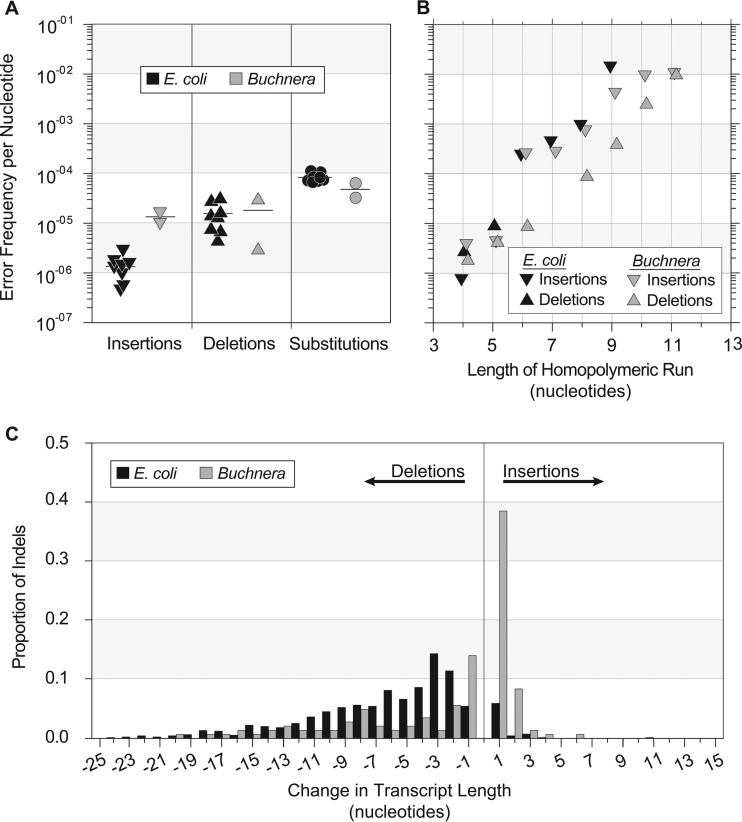
Characteristics of transcription errors in bacterial genomes. (A) Rates of transcription insertions, deletions, and base substitutions in *E. coli* and *Buchnera*. Frequencies of each type of transcription error were computed for the same eight replicate samples of *E. coli* and for the same two replicate samples of *Buchnera*. (B) Error frequencies of *Buchnera* transcription insertions, *Buchnera* transcription deletions, and *E. coli* transcription insertions in homopolymeric runs. The *y*-axes follow those of panel A. Each error type shown follows a natural exponential function (*Buchnera*_insertions_, *r*^*2*^ = 0.739, *P* < 0.004; *Buchnera*_deletions_, *r*^*2*^ = 0.981, *P* < 0.001; *E. coli*_insertions_, *r*^*2*^ = 0.894, *P* < 0.003). (There were too few *E. coli* transcription deletions to test for this trend.) (C) Length distribution of transcription insertions and deletions in *E. coli* and *Buchnera*.

We analyzed the spectrum of insertion and deletion errors across all coding regions of the transcriptomes of *Escherichia coli* and *Buchnera aphidicola* by applying a circularization method that prevents the inclusion of sequencing artifacts (15). For the eight replicate samples of *E. coli*, transcription errors causing deletions vastly outnumbered those causing insertions, 921 to 72 (see Data Set S1 and S2 in the supplemental material), yielding average rates of 1.57 × 10^−5^ deletion events and 1.35 × 10^−6^ insertion events per transcribed nucleotide (Fig. 1A). In *Buchnera*, however, the preponderance of transcription indels were insertions; across the two replicates, there was a total of 157 insertions and 70 deletions (Data Set S3 and S4), representing 1.30 × 10^−5^ insertion events and 1.75 × 10^−5^ deletion events per transcribed nucleotide. (The mean insertion rate is higher than the mean deletion rate in *Buchnera* due to the high variance among samples [Fig. 1A].) Despite their contrasting patterns, the overall rates of transcription indels in *E. coli* and *Buchnera* differed by less than 2-fold, and in *E. coli* the overall rate of transcription indels was within the same order of magnitude as transcription errors that result in base substitutions (Fig. 1A). Considering both nucleotide substitutions and indels, the cumulative transcription error rate per transcribed nucleotide was 9.94 × 10^−5^ in *E. coli* and 7.73 × 10^−5^ in *Buchnera*. We found no effects of transcript expression level, gene orientation, or error location within a transcript on the transcription indel rates in either *E. coli* or *Buchnera*.

10.1128/mBio.01230-17.2 DATA SET S1  *E. coli* transcription deletions used in our analyses. Column 1 contains the SRA accession number of the source transcriptome, column 2 indicates the biological replicate, column 3 provides the genomic location of the deletion, column 4 provides the nine bases directly upstream of the transcription deletion, column 5 provides the deleted sequence, and column 6 provides the sequence immediately downstream of the transcription deletion. Download DATA SET S1, XLSX file, 0.1 MB. Copyright © 2017 Traverse and Ochman. 2017 Traverse and Ochman This content is distributed under the terms of the Creative Commons Attribution 4.0 International license.

10.1128/mBio.01230-17.3 DATA SET S2  *E. coli* transcription insertion used in our analyses. Column 1 contains the SRA accession number of the source transcriptome, column 2 indicates the biological replicate, column 3 provides the genomic location of the insertion, column 4 provides the nine bases directly upstream of the transcription insertion, column 5 provides the inserted sequence, and column 6 provides the sequence immediately downstream of the transcription insertion. Download DATA SET S2, XLSX file, 0.01 MB. Copyright © 2017 Traverse and Ochman. 2017 Traverse and Ochman This content is distributed under the terms of the Creative Commons Attribution 4.0 International license.

10.1128/mBio.01230-17.4 DATA SET S3  *Buchnera* transcription deletions used in our analyses. Column 1 contains the SRA accession number of the source transcriptome, column 2 indicates the biological replicate, column 3 provides the genomic location of the deletion, column 4 provides the nine bases directly upstream of the transcription deletion, column 5 provides the deleted sequence, and column 6 provides the sequence immediately downstream of the transcription deletion. Download DATA SET S3, XLSX file, 0.01 MB. Copyright © 2017 Traverse and Ochman. 2017 Traverse and Ochman This content is distributed under the terms of the Creative Commons Attribution 4.0 International license.

10.1128/mBio.01230-17.5 DATA SET S4  *Buchnera* transcription insertions used in our analyses. Column 1 contains the SRA accession number of the source transcriptome, column 2 indicates the biological replicate, column 3 provides the genomic location of the insertion, column 4 provides the nine bases directly upstream of the transcription insertion, column 5 provides the inserted sequence, and column 6 provides the sequence immediately downstream of the transcription insertion. Download DATA SET S4, XLSX file, 0.02 MB. Copyright © 2017 Traverse and Ochman. 2017 Traverse and Ochman This content is distributed under the terms of the Creative Commons Attribution 4.0 International license.

### Insertion errors in homopolymeric runs.

Among the insertions that occur during transcription, 80% involve the addition of an individual nucleotide (Fig. 1C). These single-nucleotide insertions predominate in homopolymeric runs, and their frequencies increase exponentially with the length of the homopolymeric run (up to the maximum of 9 nucleotides (nt) in *E. coli* and 11 nt in *Buchnera* [Fig. 1B]). In every case, the inserted nucleotide matches those comprising the repeat, suggesting that these errors arise through a backward slippage mechanism.

The 10-fold difference in the numbers of transcription insertions in *E. coli* and *Buchnera* can be ascribed almost entirely to the incidence of homopolymeric runs in these genomes. The low (26%) G+C content of the *Buchnera* genome increases the likelihood and lengths of homopolymeric runs of adenine or thymine, which comprise 80% of the sequenced homopolymeric runs in the *E. coli* transcriptome and 97% of the sequenced homopolymeric runs in the *Buchnera* transcriptome. In both organisms, 100% of insertions within homopolymeric runs occurred in runs of adenine or thymine, indicating that homopolymeric runs of guanine and cytosine are not prone to slippage. A minority of transcription insertions (19 in *E. coli* and 6 in *Buchnera*) did not occur in these repeat tracts, but in 15 of 19 such cases in *E. coli*, the inserted nucleotide(s) matched the preceding nucleotide, suggesting that they originated by backward slippage followed by retranscription of the slipped region (Table 1).

**TABLE 1:** *E. coli* transcription insertions in nonhomopolymeric regions

Preceding sequence^a^^,^^b^	Inserted sequence^b^	Succeeding sequence^a^	Insertion length (nt)
***CGCTGGCGC***	***GCCGCTGGCGC***^c^	AATGGATAG	11
T***ATTTATTT***	***ATTT***	CGCCCTGCC	4
GTG***ATGATG***	***ATG***	TATAACCGG	3
***AGAAGAAGA***	***AGA***	TAAAAACAG	3
***TTCTTCTTC***	***TTC***	GCGAAGCGT	3
CTC***TTCTTC***	***TTC***	CAGCGTCGG	3
CTTGAG***CCG***	***CCG***	TCGTCGTGG	3
***TTCTTCTTC***	***TTC***	AACACCGAC	3
***AACAACAAC***	***AAC***	CGATGAACT	3
CGGTCTGGA	AG	CAAAGGCAC	2
CGGCGGTT***A***	***A***	TTTTTTTGC	1
TCGAAGAA***C***	***C***	GCGTTAAGA	1
TCCGTTCT***A***	***A***	CAAACATTT	1
GAACAGGC***G***	***G***	AAAAAAGTG	1
CTGAAAG***AA***	***A***	GCGGCAGAA	1
TTCGTAG***AA***	***A***	GCTGAGTAA	1
CATACCACC	T	ATCGTTAAG	1
CTGGCAGAA	G	ACGTTATCC	1
ACTGGCGGC	A	GCAAACCGG	1

a Columns list the nine preceding and the nine succeeding nucleotides, because this number corresponds to the length of RNA:DNA hybrids in the elongation complex.

b Bold italicized sequence portions represent instances of slippage followed by retranscription of the slipped region.

c In this case, the inserted nucleotides matched 11 of the preceding nucleotides, but only 9 are listed in the preceding nucleotides column.

### Transcription deletions in *E. coli* often preserve the reading frame.

In contrast to transcription insertions, the majority of transcription deletions entail multiple nucleotides. The spectra of transcription deletions differ in *E. coli* and *Buchnera*, likely stemming from their differences in nucleotide composition. In *E. coli*, trinucleotide deletions, which keep the protein in frame, are more frequent than either mono- or dinucleotide deletions (Fig. 1C), and deletions within homopolymeric tracts are rare. Additionally, there is a 3-nucleotide periodicity in short deletions of 6 or fewer bases, with peaks at 3 and 6 nucleotides in length (*P* < 0.01, Fisher exact test). In *Buchnera*, the most common transcription deletion involves single nucleotides (Fig. 1C), over half of which occur in homopolymeric tracts, but only 16 of the 57 deletions in *Buchnera* occurred within homopolymeric tracts, as opposed to 151 of the 157 insertions. The error rate of *Buchnera* deletions in homopolymeric runs increases exponentially as the length of the run increases, similar to what was observed for insertions (Fig. 1B).

### Transcription deletions are A+U biased.

**FIG 2:**
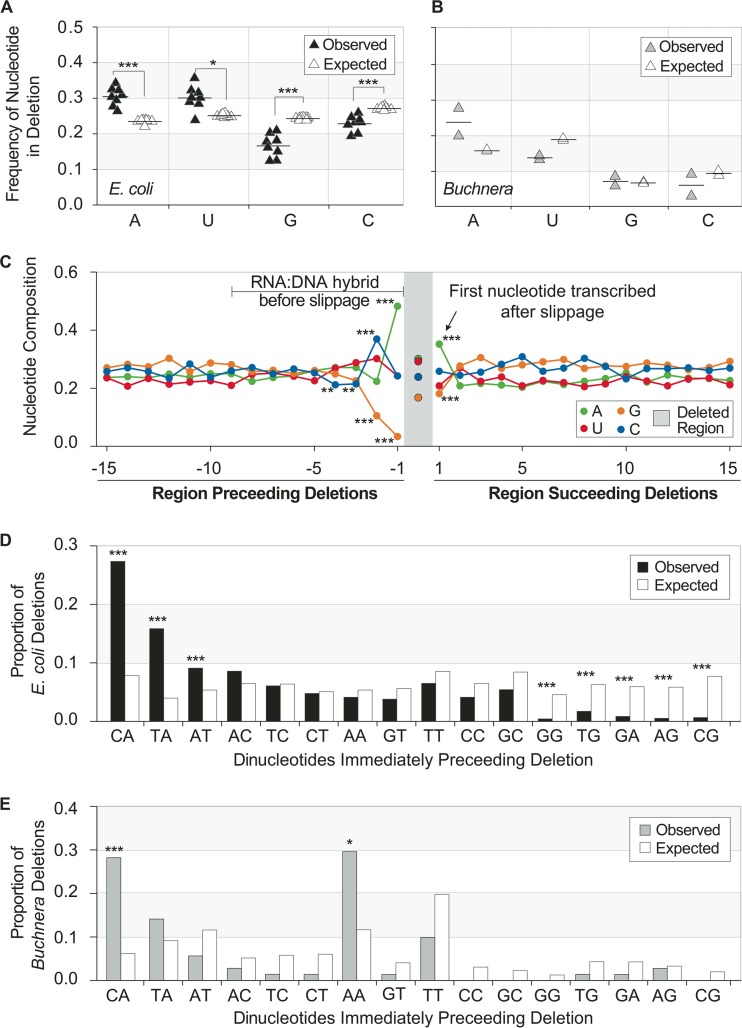
Compositional biases of transcription deletions. (A) Nucleotide composition of transcription deletions in *E. coli* (black) compared to that expected based on the nucleotide composition of all transcribed sequences (white) in each replicate. Comparisons were performed using pairwise Wilcoxon tests and data were subjected to the Benjamini-Hochberg correction. *, *P* < 0.05; ***, *P* < 0.001. (B) Nucleotide composition of transcription deletions in *Buchnera* (gray) compared to that expected based on the nucleotide composition of all transcribed sequences (white) in each replicate. Comparisons were performed using a Student’s *t*-test. The *y*-axes follow those in panel A. (C) Average proportion of each nucleotide at each of the 15 bases preceding and the 15 bases succeeding *E. coli* transcription deletions and in deletions as a whole (shaded gray region). Significant biases in nucleotide frequencies occur in the four bases before a deletion and one base after a deletion. (D) Dinucleotide frequencies of the two bases preceding transcription deletions in *E. coli* (black) compared to that expected based on the nucleotide composition of all transcribed sequences (white). (E) Dinucleotide frequencies of the two bases preceding transcription deletions in *Buchnera* (gray) compared to that expected based on the nucleotide composition of all transcribed sequences (white). Comparisons in panels C, D, and E were made using the Fisher exact test and data were subjected to the Benjamini-Hochberg correction. *, *P* < 0.05; ***, *P* < 0.001.

Within *E. coli*, there is a bias in the composition of nucleotides removed by transcription deletions. The average composition of deleted nucleotides is 39.5% G+C, differing significantly from the overall nucleotide composition of 53.3% G+C for coding regions of the transcriptome (pairwise Wilcoxon test, *P* < 0.001). Moreover, guanine and cytosine are significantly underrepresented within transcription deletions (Fig. 2A), indicating that certain nucleotide-enriched regions are resistant to slippage. Unlike *E. coli*, the nucleotide contents of transcription deletions in *Buchnera* did not differ significantly from that of the entire transcriptome (Fig. 2B), perhaps due to the already elevated A+U content of the *Buchnera* genome.

### Effects of preceding and succeeding nucleotides on transcription deletions.

Because backward slippage, as provoked by certain upstream nucleotides, was found to be a major source of transcription insertions, we asked if there were any nucleotide compositional biases in the regions preceding and succeeding each deletion (Fig. 2C). The −1 positions, i.e., the last nucleotides transcribed before slippage, were significantly enriched in adenine and deficient in guanine. The −2 positions of regions preceding deletions were significantly enriched in cytosine and were again deficient in guanine, and the −3 and −4 positions had significantly lower cytosine compositions. Additionally, the +1 position, i.e., the first nucleotide transcribed after a deletion, was significantly enriched in adenine and had a significantly lower guanine composition.

The nucleotide composition of dinucleotides surrounding transcription indels was also biased: transcription deletions in *E. coli* were more likely to occur immediately after transcription of CA, TA, or AT dinucleotides (Fisher’s exact test, *P* < 0.001), whereas many of the G-rich dinucleotides (GG, TG, GA, AG, and CG) were less likely to promote a deletion (*P* < 0.001) (Fig. 2D). The dinucleotide composition of regions further upstream did not impose any detectable effect on the occurrence of transcription deletions. Although there were insufficient deletions in *Buchnera* to test the influences of all dinucleotide pairs, transcription deletions occurred at significantly higher frequencies when CA or AA was the preceding dinucleotide (Fig. 2E). All significantly higher or lower incidences of trinucleotides could be explained by the trends observed for dinucleotides.

### Slippage stops at locations with high RNA:DNA hybrid complementarity.

In the current model for transcription deletions, the elongation complex and transcript lose register with the template DNA (such that the transcript and DNA template are no longer paired), slip forward, and then resume transcription at a downstream point on the template DNA (19). After a slippage event, the RNA:DNA hybrid between the nine most recently transcribed nucleotides and the DNA template commonly contains several mismatches, because the elongation complex resides in a new location.

**FIG 3:**
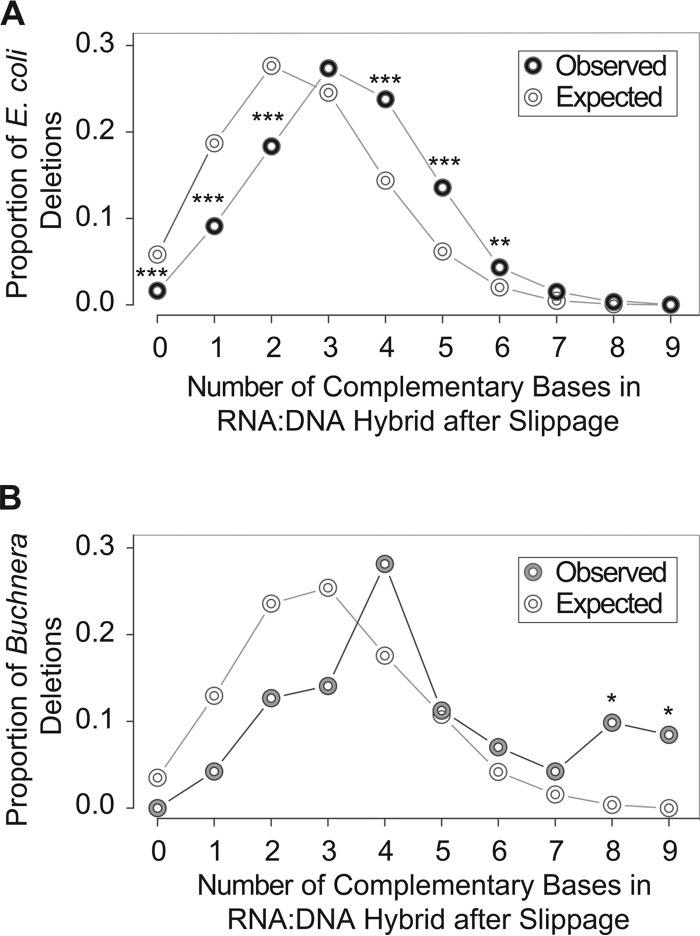
Dependence of transcription deletions on sequence complementarity in the RNA:DNA hybrid. (A) The 9-base RNA:DNA hybrids were reconstructed for transcription deletions (black rings) and for expected deletions based on the nucleotide composition of all transcribed sequences (white rings) in *E. coli*, and the extent of complementarity between the region preceding the end of a deletion and the RNA:DNA hybrids was computed. (B) The 9-base RNA:DNA hybrids were reconstructed for transcription deletions (gray rings) and for deletions expected based on the nucleotide composition of all transcribed sequences (white rings) in *Buchnera*, and the extent of complementarity between the region preceding the end of a deletion and the RNA:DNA hybrids was computed. Due to the small sample size with *Buchnera*, only 8 and 9 bases of RNA:DNA hybrid complementarity were significant. For both organisms, there were significant deviations from expectation (chi-square test, *P* < 0.001), indicating that transcription slippage is more likely to stop at regions of higher base complementarity than expected. Comparisons of the extent of RNA:DNA complementarity were performed using the Fisher exact test and data were subjected to the Benjamini-Hochberg correction. *, *P* < 0.05; **, *P* < 0.01; ***, *P* < 0.001.

To calculate the extent of complementarity between the slipped transcript and the new DNA template location, we reconstructed the RNA:DNA hybrids after slippage by comparing the nine nucleotides immediately preceding the start of each deletion with the nine nucleotides preceding the end of each deletion, each from the annotated start and endpoints of the deletion in the reference sequence. In both *E. coli* and *Buchnera*, the reconstructed RNA:DNA hybrids from observed deletions had more complementary base pairing than expected (Fig. 3A and B) (chi-square tests, *P* < 0.0001), indicating that after a slippage event, transcription is more likely to resume in regions that impart high complementarity within the new RNA:DNA hybrid.

### Transcriptional deletions are associated with sequence repeats in *E. coli*.

**FIG 4:**
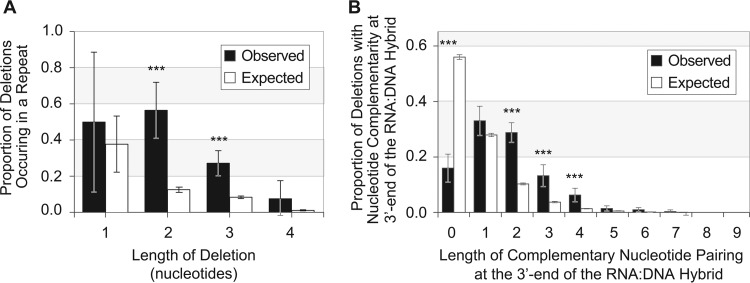
Transcription deletions in short sequence repeats. (A) Proportions of transcription deletions between 1 and 4 nucleotides in length occurring within repetitive sequences in *E. coli*. In all cases, deletion lengths correspond to the length of the repeat unit within a repetitive sequence, and there is a minimum of two repeat units for a sequence to be considered repetitive. (The wide error bars in single nucleotide deletions result from replicates with few or no deletions of that length.) (B) Proportions of deletions with successive complementary bases in the 3′ end of the RNA:DNA hybrid after slippage. Deletions of all lengths were included in this analysis. Comparisons in panels A and B were performed with pairwise Wilcoxon tests (*n* = 8 for each test), and data were subjected to the Benjamini-Hochberg correction. *, *P* < 0.05; **, *P* < 0.01; ***, *P* < 0.001.

We next examined the extent to which nonhomopolymeric repeat sequences were associated with transcriptional deletions. Deletions of two or three nucleotides were significantly more likely to occur in regions containing di- or trinucleotide repeats, respectively (Fig. 4A) (Wilcoxon test, *P* < 0.01). Overall, about half of all two-nucleotide deletions occurred in a dinucleotide repeat, and 37 of the 143 three-nucleotide deletions occurred in a trinucleotide repeat. Unlike insertions, these short deletions did not increase in frequency with repeat number: 34 of the 37 repeating runs that promoted deletions consisted of only two repeats, with the second instance of the repeat experiencing the deletion.

To determine if deletions are more likely to occur when repeats are separated by intervening sequences, we enumerated the deletions that were complementary to the new DNA template at the 3′ end of the RNA portion of the RNA:DNA hybrid. Nearly 30% (*n* = 265) of all deletions had complementary base pairing in the last two positions in the RNA:DNA hybrid, a percentage significantly higher than that expected by chance (Fig. 4B). Additionally, there was an increased occurrence of complementary base pairing of all nucleotides within the last three and four positions of the slipped transcript and the new DNA template (Wilcoxon test, *P* < 0.01) (Fig. 4B). In sum, the final two, three, or four positions in RNA:DNA hybrids are significantly more likely to experience complementary base pairing after forward transcription slippage.

## DISCUSSION

Previous studies of the insertions and deletions that arise during transcription examined only those errors that occurred within long synthetic homopolymeric repeats, and the reported findings provided neither the absolute rate of transcription indels nor the full spectrum of sequence motifs prone to such errors (17–20). These issues can now be resolved through the application of a genome-wide approach that assays errors incurred over the entire transcriptome and furnishes accurate estimates of error rates based on the actual number of nucleotides transcribed.

Cumulatively, there were 993 indels (921 deletions and 72 insertions) in *E. coli* and 227 indels (70 deletions and 157 insertions) in *Buchnera*, yielding rates of 1.7 × 10^−5^ and 3.1 × 10^−5^ indels per transcribed nucleotide, respectively. The transcription error rates for indels were several-fold lower, but within the same order of magnitude as the transcription error rate for base substitutions reported for *E. coli* and *Buchnera* (4), yielding overall transcription error rates of nearly 10^−4^ per transcribed nucleotide. Given an average gene length in bacteria of 10^3^ bp, this indicates that 1 in 10 transcripts have some type of transcription error. Such high rates can be tolerated because transcription errors, in contrast to replication errors, are ephemeral and usually affect only a very small fraction of the proteins produced from a given locus; additionally, there are mechanisms to refold and remove damaged proteins (12, 23, 24). The strong mutational bias of *Buchnera* toward A+T promotes the occurrence of long homopolymeric tracts, which experience frequent indels during replication, giving rise to pseudogenes (25, 26). It has been proposed that transcription indels might serve to correct these frameshifted pseudogenes (26); however, we detected no cases where a transcription error restored a reading frame.

Based on early models of transcription slippage, previous assays of transcription indels were designed to detect errors occurring in homopolymeric runs. With respect to transcription insertions, our results largely corroborate prior findings, because our genome-wide approach showed that in both *E. coli* and *Buchnera*, a majority of transcription insertions involve the addition of a single base into homopolymeric runs of either A or T (17–20). Those few insertions (18 in *E. coli*, 3 in *Buchnera*) that occurred in nonhomopolymeric sequences—errors that were previously never assayed—mostly involved duplications of the preceding nucleotide, suggesting that virtually all transcription insertions, whether in homopolymers or not, are caused by retranscription after an event of backward slippage.

In contrast to transcription insertions, most transcription deletions occurred in sequences that are more complex and were therefore missed by previous assays, which focused solely on transcription errors in homopolymeric runs. Only 15 (21%) transcription deletions in *Buchnera* were initiated within uninterrupted homopolymeric runs, and only 3 (less than 1%) transcription deletions in *E. coli* were initiated within uninterrupted homopolymeric runs, a difference likely attributable to the very high incidence of homopolymeric runs in *Buchnera*.

Although the relatively high frequencies of transcription errors compared to replication errors imply that transcription errors are generally of little consequence to cellular fitness, we detected a 3-nt periodicity in deletions of 6 or fewer nucleotides in *E. coli*, suggesting that selection serves to avoid or eliminate frameshifting deletions. Transcription deletions are common in *E. coli*, and these ≤6-nt deletions comprise 58% of all deletions in *E. coli*, so it possible that they occur at frequencies high enough to impact fitness. Despite similarities in the rates of transcription deletions in *E. coli* and *Buchnera*, the periodicity in transcription deletions was not apparent in *Buchnera*, most likely because selection is less effective due to the small effective population sizes.

Knowledge of the full scope of transcription errors provides several insights into the mechanisms by which transcription indels arise. In brief, most deletions were greater than 1 nucleotide in length, whereas most insertions were 1 nucleotide in length, arising from the backward slippage of the elongation complex by only 1 base before transcription resumes (Fig. 1). Because multiple elongation complexes can transcribe genes in arrays (27, 28), it is possible that once the nascent transcript loses register with the DNA template and the elongation complex slips backwards, upstream elongation complexes push the slipped elongation complex forward, thereby limiting how far back it can slip. Since the vast majority of deletions are greater than 1 nucleotide in length (Fig. 1C), following this scenario, it appears that the upstream elongation complexes can propel the slipping elongation complex, causing it to skip forward several nucleotides.

A process by which upstream arrays of elongation complexes (i) prevent large insertions by blocking further backward slippage, (ii) help restore the original position of a slipped elongation complex, and (iii) facilitate translocation to distant positions helps to explain, based on all three of these aspects of the process, the low insertion-to-deletion rate in *E. coli*. Once an elongation complex and transcript lose register with the template and slip backward, the forward translocation from an array of actively transcribing elongation complexes will most likely result in a deletion rather than an insertion. This process likely operates in *Buchnera* as well, but the high incidence of homopolymeric runs makes for many more backward slippage events, thereby elevating the number of insertions.

Although the majority of the transcription insertions originate in homopolymeric runs, several insertions occurred outside these sequences, suggesting that several mechanistically similar events cause transcription insertions in both *E. coli* and *Buchnera*: (i) for insertions at homopolymeric runs, a backward slip of the elongation complex at these sites usually retains complementary base pairing between the 3′ end of the transcript and the template DNA, allowing transcription to resume because the template sequence before and after the slippage event remains identical. (ii) For those insertions occurring at tri- or tetranucleotide repeats, the elongation complex slipped backwards by one repeat and then transcribed an extra repeat (Table 1), again retaining complementary base pairing within a portion of the RNA:DNA hybrid, similar to slippage in homopolymeric regions. (iii) Of the 11 *E. coli* insertions that did not occur in repeat regions, 7 could be explained by backward slippage followed by retranscription of the slipped bases; for the remaining 4 insertions in which the inserted nucleotides did not match the bases preceding the insertion, there were no sequence characteristics that signified the source of the error.

Unlike backward slippage events, the preponderance of which occurred in runs of A or T, forward slippage events, which result in transcription deletions, were not dependent on homopolymeric repeats and were significantly more likely to occur when the most recently transcribed two bases were CA, UA, or AU in *E. coli*. Additionally, our finding that guanine is underrepresented before and after a transcription deletion aligns with a finding that guanine is enriched at the −2 and +1 sites in pause-prone sequences (29), implying that G-rich sequences stimulate pausing, whereas G-poor sequences are slippery.

These new transcriptome-wide data support a revised model of transcription slippage in which increased RNA:DNA hybrid complementarity after slippage fosters the elongation complex to resume transcription at a new site, resulting in a transcription insertion or deletion. Previous models implied that the occurrence of transcription slippage was limited to homopolymeric runs; we now conclude that it is the overall complementarity of the RNA:DNA hybrid after transcription slippage that contributes to the creation of indels.

When a ribonucleotide is misincorporated during transcription, the unpaired base causes the transcript to bend away from the template (“fraying” [39]), which induces the elongation complex to pause and translocate backwards while extruding the unpaired base, a process termed “backtracking” (30, 31). If a transcription slippage event results in mispairing at the 3′ end of the RNA:DNA hybrid, it may resemble a misincorporation, causing the elongation complex to attempt to backtrack. Because the transcript and elongation complex reside in a new location after slippage, backtracking will be blocked because this process requires complementary base pairing between the transcript and the DNA template (30, 31). If a portion of the nascent transcript slips forward and through the elongation complex before slippage stops, the resulting RNA:DNA hybrid can resemble a backtracked state, such that nucleolytic cleavage might still occur. However, the orientation of nascent transcripts relative to the elongation complex cannot be inferred from our assays, so the effect of nucleolytic cleavage on slipped transcripts presently remains unknown.

Because the stability of the elongation complex is affected by the RNA:DNA hybrid, low complementarity after slippage may cause the slipped elongation complex to dissociate (20, 30, 32). However, if upstream elongation complexes collide with the slipped elongation complex before it dissociates due to poor base complementarity, it may be advanced forward to a region of high RNA:DNA hybrid complementarity so that transcription can resume. If the forward action of upstream elongation complexes is the primary mechanism of forward slippage, the distance that an elongation complex can be pushed before it dissociates may dictate the maximum length of transcription deletions.

The mechanisms that generate indels during replication are similar to those that result in transcription slippage, but there are fundamental differences between the processes. First, the majority of indels in DNA occur in short repetitive regions (33), whereas those in RNA transcripts occur in more complex sequences. In DNA, indels are thought to be generated by slipped-strand misalignment (33), and our model of transcription indels involves a similar mechanism but does not require the presence of direct repeats. Second, small indels in DNA can be generated through deoxynucleoside triphosphate-stabilized misalignment (34), whereas a similar mechanism occurring during RNA transcription would produce base substitutions. The difference is due to the manner by which DNA polymerase and RNA polymerase handle the conformational constraints of a displaced base (35, 36).

**FIG 5:**
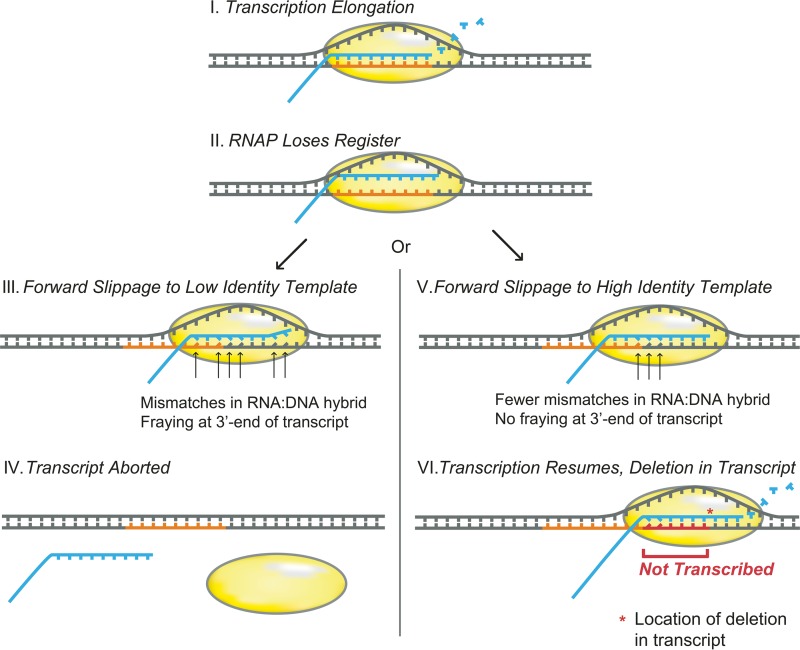
Model of transcription slippage resulting in deletions. Based on locations and sequence contents of deletions genome-wide, the degree of complementarity of the RNA:DNA hybrid after a transcription slippage event (I and II) determines whether transcription is aborted, producing a truncated transcript (III and IV), or resumed, producing a transcript containing a deletion (V and VI). Steps in the model use the following notations: template DNA is shown in black, transcript RNA and incoming ribonucleotides are in blue, the original RNA:DNA hybrid location is orange, the nontranscribed (i.e., deleted) region is shown in red, mismatched bases are shown as angled contacts between noncomplementary nucleotides, and the RNA polymerase transcription elongation complex is represented by a yellow bubble. In this model, normal transcription (I) becomes interrupted when the elongation complex and transcript lose register with the DNA template (II). Possible outcomes include the elongation complex slipping forward to a region of low complementarity (III), and in the example depicted, the elongation complex slips forward 5 bases, landing on a template location where 6 of the 9 bases in the RNA:DNA hybrid are not complementary. If transcription cannot resume due to the extent of mispairing in the RNA:DNA hybrid and/or fraying at the end of the transcript, the transcript is aborted (IV). Alternatively, if the elongation complex slips to template location with fewer mismatches (V), the 3′ end of the RNA bonds sufficiently to the DNA template, and transcription resumes (VI) after the skipped the region, generating a deletion.

Overall, our model of transcription slippage (Fig. 5; Fig. S1) involves two steps that lead to transcription insertions and deletions. First, the transcript RNA loses register with the template DNA, causing the elongation complex to slip along the template DNA. The amount of slippage is influenced by the presence of upstream elongation complexes, which can block extensive backward slippage and even propel the slipping elongation complex to a new location. Next, slippage events that result in high RNA:DNA hybrid complementarity, particularly at the 3′ end, lead to reinitiation of transcription elongation to generate an insertion or deletion. Whereas our model is based on the sequence locations at which indels occur, additional experimental work is required to determine the accuracy of the proposed mechanism.

10.1128/mBio.01230-17.1 FIG S1  Model of transcription slippage and the resulting insertions. Based on locations and sequence contents of insertions genome-wide, the degree of complementarity of the RNA:DNA hybrid after a transcription slippage event (I and II) determines whether transcription is aborted, produces a truncated transcript (III and IV), or resumes, producing a transcript containing an insertion (V and VI). Steps in the model use the following notations: black, template DNA; blue, transcript RNA and incoming ribonucleotides; orange, the original RNA:DNA hybrid location; green, the retranscribed (i.e., insertion) region; mismatched bases are shown as angled contacts between noncomplementary nucleotides, and the RNA polymerase transcription elongation complex is represented by a yellow bubble. In this model, normal transcription (I) becomes interrupted when the elongation complex and transcript lose register with the DNA template (II). Possible outcomes include the elongation complex slipping backward to a region of low complementarity (III), and in the example depicted, the elongation complex slips backward 1 base, landing on a template location where 6 of the 9 bases in the RNA:DNA hybrid are not complementary. If transcription cannot resume due to the extent of mispairing in the RNA:DNA hybrid and/or fraying at the end of the transcript, the transcript is aborted (IV). Alternatively, if the elongation complex slips to template location with fewer mismatches, in this case a homopolymeric run (V), the 3′ end of the RNA bonds sufficiently to the DNA template, and transcription resumes (VI) after the retranscribed region, generating an insertion. Download FIG S1, PDF file, 1.8 MB. Copyright © 2017 Traverse and Ochman. 2017 Traverse and Ochman This content is distributed under the terms of the Creative Commons Attribution 4.0 International license.

## MATERIALS AND METHODS

### Strain information, sequencing procedures, and detection of indels.

We assayed the transcriptomes of eight biological replicates of *Escherichia coli* MG1655 and two biological replicates of *Buchnera aphidicola* LSR1 by using the CirSeq library preparation protocol (15). In this method, mRNA is sheared into 80- to 100-bp fragments, which are then circularized, primed using random hexamers, and reversed transcribed to generate cDNA that contains multiple linked repeats of the mRNA fragment. cDNAs containing these repeats were sequenced using Illumina MiSeq 300-nt read lengths to capture at least three repeats within a sequencing read. Reads were processed by the CirSeq_v3 pipeline (http://andino.ucsf.edu/CirSeq) to generate a consensus sequence for each read (14). All settings used in CirSeq_v3 were the default settings, with a quality score cutoff of 20. CirSeq_v3 uses Bowtie 2 (37) to align reads to a reference genome (NC_000913.3 for *E. coli* and NZ_ACFK01000001 for *Buchnera*). Additionally, we edited the run.sh script to retain the intermediate output (9_alignment.sam and 10_alignment.sam) generated in the CirSeq_v3 pipeline, since these outputs contain candidate insertions and deletions. Additional strain information and library preparation protocols have been described elsewhere (4). The data are publicly available from the NCBI Sequence Read Archive (SRA) [see “Accession number(s),” below]. (The insertions and deletions used in our analyses are provided as Data Sets in the supplemental material.)

By generating a consensus sequence from the multiple repeats within a single read, sequencing errors, which appear as changes in only one of the repeats, are omitted. Insertion and deletion rates of *Illumina* sequencing are very low (38), and only those insertions or deletions that occurred at identical positions and are of equal size in fully aligned repeats were considered authentic. Because sequencing reads originate from the reverse transcription of circularized mRNA fragments primed with random hexamers, the actual orientation of sequences can only be determined after multiple rounds of sequence alignment. This process generates many intermediate alignment files (9_alignment.sam and 10_alignment.sam) that contain improperly mapped reads, and to detect insertions and deletions, we searched these files to identify reads that contained indels flanked on both sides by fully aligned sequences. One strategy for determining the correct orientation of a read in the CirSeq_v3 pipeline was to sequentially move each base from one end of the read to the other (14). By mapping each iteration to the genome, many reads that initially contained insertions or deletions eventually yielded an aligned sequence devoid of indels. To identify insertions and deletions, we retained those reads that contained the highest alignment score within each iteration of a read while also containing an insertion or deletion. Finally, only those insertions receiving quality scores of ≥20 and only those deletions that were flanked on both sides by bases receiving quality scores of ≥20 were considered. Additionally, we sequenced the genome of the parental strain of *E. coli* to confirm that no errors were attributable to genomic mutations. Statistical analyses were performed with Prism GraphPad and R.

### Simulations.

To determine if the observed deletions were biased toward specific sequences, we calculated their expected occurrence through simulations based on the frequencies of gene transcripts in the transcriptome. The average read depth of each gene was tabulated, and genes were sampled at random, weighted by read depths. Because there was no observed bias in the locations of deletions within genes, simulated deletions could be allowed to randomly occur anywhere within the coding region of a transcript. The length of each deletion was drawn from the distribution of deletion lengths for each replicate without resampling. We performed 100 replicate simulations for each transcriptome examined. All simulations were subjected to the same adjustments and analyses (described below) as the observed deletions.

### Ascertaining locations and contents of deletions

In many cases, it is possible to identify the precise location of a deletion by aligning reads to the reference sequence; however, many deletions occur in regions of low sequence complexity or involved the deletion of a repeat in a repetitive sequence. Such cases can result in ambiguities in ascertaining which of the multiple, identical repeat units has been deleted, and so these were resolved by positioning the ambiguous portion to the 3′ end of the deletion. Because this procedure may artificially increase base complementarity at the 3′ end of reconstructed RNA:DNA hybrids (see below), we controlled for any introduced biases by treating simulated deletions in the same manner.

### Computing indel rates.

Transcriptome-wide rates of insertions and deletions of *E. coli* and *Buchnera* were calculated for each replicate by dividing the total number of insertions or deletions in protein-coding transcripts by the sequencing coverage of the corresponding regions and averaging across replicates. To calculate the indel error rates at homopolymeric runs, we first identified all homopolymeric runs of ≥4 nucleotides in length within protein-coding genes of *E. coli* MG1655 and *B. aphidicola* LSR1, and then we determined the numbers of insertions and deletions originating in runs of each length category. When evaluating error rates in homopolymeric runs, or across any gene category, indel frequencies were normalized to the sequence coverage for each category. To determine the effect of transcript abundance on error rate, all genes were binned by their average coverage, and the errors and total coverage were tabulated for each bin. Coverage bins increased in 1× increments from 0- to 10-fold coverage, 10× increments from 10- to 100-fold coverage; 100× increments from 100- to 500-fold coverage, and subsequently in 500- to 1,000-fold, 1,000- to 2,000-fold, and >2,000-fold coverage bins. Transcriptomes were analyzed using custom python scripts, and all statistics were performed using Prism GraphPad and R.

### Features of deleted regions.

The nucleotide compositions of deleted nucleotides and of the 15-bp regions preceding and succeeding each deletion were calculated by direct count for each observed or simulated replicate and then pooled across replicates. We inferred the complementarity of bases within the RNA:DNA hybrids after a slippage event by comparing the nine nucleotides directly preceding the start of each deletion to the nine nucleotides directly preceding the end of each deletion. The nine nucleotides preceding the start of each deletion represent the nucleotides transcribed before the slippage event and constitute the RNA portion of the RNA:DNA hybrid, and the nine nucleotides preceding the end of each deletion represent the region in which slippage stopped and thus constitute the new portion of DNA in the RNA:DNA hybrid.

### Accession number(s).

The sequencing data are publicly available from the NCBI SRA (SRP072992). The following individual accession numbers for sequences were assigned: SRX1694197, SRX1694194, SRX1694017, SRX1694016, SRX1694007, SRX1694003, SRX1693946, SRX1693944, SRX1686622, and SRX1686515.

## References

[1] Rosenberger RF, Foskett G 1981 An estimate of the frequency of *in vivo* transcriptional errors at a nonsense codon in *Escherichia coli*. Mol Gen Genet 183:561–563. doi:10.1007/BF00268784. 7038382

[2] Imashimizu M, Oshima T, Lubkowska L, Kashlev M 2013 Direct assessment of transcription fidelity by high-resolution RNA sequencing. Nucleic Acids Res 41:9090–9104. doi:10.1093/nar/gkt698. 23925128 PMC3799451

[3] Yuzenkova Y, Gamba P, Herber M, Attaiech L, Shafeeq S, Kuipers OP, Klumpp S, Zenkin N, Veening JW 2014 Control of transcription elongation by GreA determines rate of gene expression in *Streptococcus pneumoniae*. Nucleic Acids Res 42:10987–10999. doi:10.1093/nar/gku790. 25190458 PMC4176173

[4] Traverse CC, Ochman H 2016 Conserved rates and patterns of transcription errors across bacterial growth states and lifestyles. Proc Natl Acad Sci U S A 113:3311–3316. doi:10.1073/pnas.1525329113. 26884158 PMC4812759

[5] Tehranchi AK, Blankschien MD, Zhang Y, Halliday JA, Srivatsan A, Peng J, Herman C, Wang JD 2010 The transcription factor DksA prevents conflicts between DNA replication and transcription machinery. Cell 141:595–605. doi:10.1016/j.cell.2010.03.036. 20478253 PMC2919171

[6] Trautinger BW, Jaktaji RP, Rusakova E, Lloyd RG 2005 RNA polymerase modulators and DNA repair activities resolve conflicts between DNA replication and transcription. Mol Cell 19:247–258. doi:10.1016/j.molcel.2005.06.004. 16039593

[7] Washburn RS, Gottesman ME 2011 Transcription termination maintains chromosome integrity. Proc Natl Acad Sci U S A 108:792–797. doi:10.1073/pnas.1009564108. 21183718 PMC3021005

[8] Dutta D, Shatalin K, Epshtein V, Gottesman ME, Nudler E 2011 Linking RNA polymerase backtracking to genome instability in *E. coli*. Cell 146:533–543. doi:10.1016/j.cell.2011.07.034. 21854980 PMC3160732

[9] D’Ari R, Casadesús J 1998 Underground metabolism. Bioessays 20:181–186. doi:10.1002/(SICI)1521-1878(199802)20:2<181::AID-BIES10>3.0.CO;2-0. 9631663

[10] Meyerovich M, Mamou G, Ben-Yehuda S 2010 Visualizing high error levels during gene expression in living bacterial cells. Proc Natl Acad Sci U S A 107:11543–11548. doi:10.1073/pnas.0912989107. 20534550 PMC2895060

[11] Gordon AJE, Satory D, Halliday JA, Herman C 2013 Heritable change caused by transient transcription errors. PLoS Genet 9:e1003595. doi:10.1371/journal.pgen.1003595. 23825966 PMC3694819

[12] Fan Y, Wu J, Ung MH, De Lay N, Cheng C, Ling J 2015 Protein mistranslation protects bacteria against oxidative stress. Nucleic Acids Res 43:1740–1748. doi:10.1093/nar/gku1404. 25578967 PMC4330365

[13] Rosenberger RF, Hilton J 1983 The frequency of transcriptional and translational errors at nonsense codons in the *lacZ* gene of *Escherichia coli*. Mol Gen Genet 191:207–212. doi:10.1007/BF00334815. 6353160

[14] Acevedo A, Brodsky L, Andino R 2014 Mutational and fitness landscapes of an RNA virus revealed through population sequencing. Nature 505:686–690. doi:10.1038/nature12861. 24284629 PMC4111796

[15] Acevedo A, Andino R 2014 Library preparation for highly accurate population sequencing of RNA viruses. Nat Protoc 9:1760–1769. doi:10.1038/nprot.2014.118. 24967624 PMC4418788

[16] James K, Gamba P, Cockell SJ, Zenkin N 2017 Misincorporation by RNA polymerase is a major source of transcription pausing *in vivo*. Nucleic Acids Res 45:1105–1113. doi:10.1093/nar/gkw969. 28180286 PMC5388426

[17] Wagner LA, Weiss RB, Driscoll R, Dunn DS, Gesteland RF 1990 Transcriptional slippage occurs during elongation at runs of adenine or thymine in *Escherichia coli*. Nucleic Acids Res 18:3529–3535. doi:10.1093/nar/18.12.3529. 2194164 PMC331007

[18] Gordon AJE, Halliday JA, Blankschien MD, Burns PA, Yatagai F, Herman C 2009 Transcriptional infidelity promotes heritable phenotypic change in a bistable gene network. PLoS Biol 7:364–370. doi:10.1371/journal.pbio.1000044. PMC2652393 19243224

[19] Zhou YN, Lubkowska L, Hui M, Court C, Chen S, Court DL, Strathern J, Jin DJ, Kashlev M 2013 Isolation and characterization of RNA polymerase *rpoB* mutations that alter transcription slippage during elongation in *Escherichia coli*. J Biol Chem 288:2700–2710. doi:10.1074/jbc.M112.429464. 23223236 PMC3554936

[20] Parks AR, Court C, Lubkowska L, Jin DJ, Kashlev M, Court DL 2014 Bacteriophage λ N protein inhibits transcription slippage by *Escherichia coli* RNA polymerase. Nucleic Acids Res 42:5823–5829. doi:10.1093/nar/gku203. 24711367 PMC4027172

[21] Coenye T, Vandamme P 2005 Characterization of mononucleotide repeats in sequenced prokaryotic genomes. DNA Res 12:221–233. doi:10.1093/dnares/dsi009. 16769685

[22] Gur-Arie R, Cohen CJ, Eitan Y, Shelef L, Hallerman EM, Kashi Y 2000 Simple sequence repeats in *Escherichia coli*: abundance, distribution, composition, and polymorphism. Genome Res 10:62–71. 10645951 PMC310497

[23] Goldberg AL 2003 Protein degradation and protection against misfolded or damaged proteins. Nature 426:895–899. doi:10.1038/nature02263. 14685250

[24] Calloni G, Chen T, Schermann SM, Chang HC, Genevaux P, Agostini F, Tartaglia GG, Hayer-Hartl M, Hartl FU 2012 DnaK functions as a central hub in the *E. coli* chaperone network. Cell Rep 1:251–264. doi:10.1016/j.celrep.2011.12.007. 22832197

[25] Moran NA, Mclaughlin HJ, Sorek R 2009 The dynamics and time scale of ongoing genomic erosion in symbiotic bacteria. Science 323:379–382. doi:10.1126/science.1167140. 19150844

[26] Tamas I, Wernegreen JJ, Nystedt B, Kauppinen SN, Darby AC, Gomez-Valero L, Lundin D, Poole AM, Andersson SG 2008 Endosymbiont gene functions impaired and rescued by polymerase infidelity at poly(A) tracts. Proc Natl Acad Sci U S A 105:14934–14939. doi:10.1073/pnas.0806554105. 18815381 PMC2567471

[27] Quan S, Zhang N, French S, Squires CL 2005 Transcriptional polarity in rRNA operons of *Escherichia coli nusA* and *nusB* mutant strains. J Bacteriol 187:1632–1638. doi:10.1128/JB.187.5.1632-1638.2005. 15716433 PMC1063997

[28] Epshtein V, Toulmé F, Rahmouni AR, Borukhov S, Nudler E 2003 Transcription through the roadblocks: the role of RNA polymerase cooperation. EMBO J 22:4719–4727. doi:10.1093/emboj/cdg452. 12970184 PMC212720

[29] Larson MH, Mooney RA, Peters JM, Windgassen T, Nayak D, Gross CA, Block SM, Greenleaf WJ, Landick R, Weissman JS 2014 A pause sequence enriched at translation start sites drives transcription dynamics *in vivo*. Science 344:1042–1047. doi:10.1126/science.1251871. 24789973 PMC4108260

[30] Nudler E, Mustaev A, Lukhtanov E, Goldfarb A 1997 The RNA-DNA hybrid maintains the register of transcription by preventing backtracking of RNA polymerase. Cell 89:33–41. doi:10.1016/S0092-8674(00)80180-4. 9094712

[31] Nudler E 2012 RNA polymerase backtracking in gene regulation and genome instability. Cell 149:1438–1445. doi:10.1016/j.cell.2012.06.003. 22726433 PMC3815583

[32] Sidorenkov I, Komissarova N, Kashlev M 1998 Crucial role of the RNA:DNA hybrid in the processivity of transcription. Mol Cell 2:55–64. doi:10.1016/S1097-2765(00)80113-6. 9702191

[33] Baptiste BA, Jacob KD, Eckert KA 2015 Genetic evidence that both dNTP-stabilized and strand slippage mechanisms may dictate DNA polymerase errors within mononucleotide microsatellites. DNA Repair 29:91–100. doi:10.1016/j.dnarep.2015.02.016. 25758780 PMC4426045

[34] Kobayashi S, Valentine MR, Pham P, O’Donnell M, Goodman MF 2002 Fidelity of *Escherichia coli* DNA polymerase IV. Preferential generation of small deletion mutations by dNTP-stabilized misalignment. J Biol Chem 277:34198–34207. doi:10.1074/jbc.M204826200. 12097328

[35] Pomerantz RT, Temiakov D, Anikin M, Vassylyev DG, McAllister WT 2006 A mechanism of nucleotide misincorporation during transcription due to template-strand misalignment. Mol Cell 24:245–255. doi:10.1016/j.molcel.2006.08.014. 17052458 PMC2810628

[36] Kashkina E, Anikin M, Brueckner F, Pomerantz RT, McAllister WT, Cramer P, Temiakov D 2006 Template misalignment in multisubunit RNA polymerases and transcription fidelity. Mol Cell 24:257–266. doi:10.1016/j.molcel.2006.10.001. 17052459

[37] Langmead B, Salzberg SL 2012 Fast gapped-read alignment with Bowtie 2. Nat Methods 9:357–359. doi:10.1038/nmeth.1923. 22388286 PMC3322381

[38] Schirmer M, D’Amore R, Ijaz UZ, Hall N, Quince C 2016 Illumina error profiles: resolving fine-scale variation in metagenomic sequencing data. BMC Bioinformatics 17:125. doi:10.1186/s12859-016-0976-y. 26968756 PMC4787001

[39] Toulokhonov I, Zhang J, Palangat M, Landick R 2007 A central role of the RNA polymerase trigger loop in active-site rearrangement during transcriptional pausing. Mol Cell 27:406–419. 17679091 10.1016/j.molcel.2007.06.008

