# Supplementary material for: Genome-Wide Spectra of Transcription Insertions and Deletions Reveal That Slippage Depends on RNA:DNA Hybrid Complementarity
Source: mBio. 2017 Aug 29;8(4):e01230-17. doi: 10.1128/mBio.01230-17 (PMC5574713; doi:10.1128/mBio.01230-17)
Supplement: FIG S1 [file mbo004173454sf1.pdf]

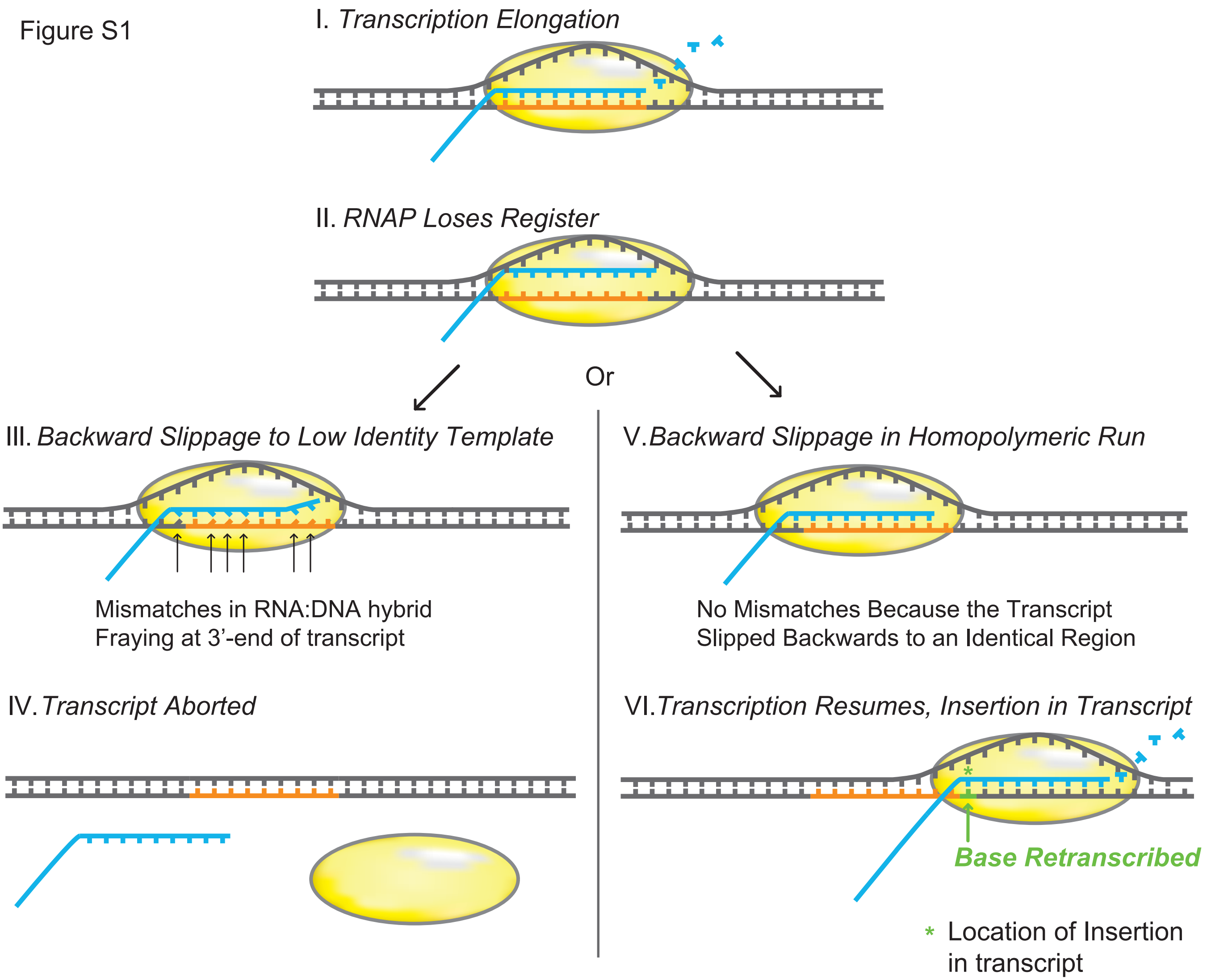
Figure S1
I. Transcription Elongation
II. RNAP Loses Register
Or
III. Backward Slippage to Low Identity Template
Mismatches in RNA:DNA hybrid
Fraying at 3'-end of transcript
IV. Transcript Aborted
V. Backward Slippage in Homopolymeric Run
No Mismatches Because the Transcript
Slipped Backwards to an Identical Region
VI. Transcription Resumes, Insertion in Transcript
Base Retranscribed
* Location of Insertion
in transcript

Figure S1. Model of transcription slippage resulting in insertions. Based on locations and sequence contents of insertions genome-wide, the degree of complementarity of the RNA:DNA hybrid after a transcription slippage event (I and II) determines whether transcription is aborted, producing a truncated transcript (III and IV), or resumed, producing a transcript containing an insertion (V and VI). Steps in the model use the following notation: Template DNA is shown in black, transcript RNA and incoming ribonucleotides in blue, the original RNA:DNA hybrid location is orange, the re-transcribed (*i.e.*, insertion) region in green, mismatched bases as angled contacts between non-complementary nucleotides, and the RNAP transcription elongation complex is represented by a yellow bubble. In this model, normal transcription (I) becomes interrupted when the elongation complex and transcript lose register with the DNA template (II). Possible outcomes include, the elongation complex slipping backward to a region of low complementarity (III), and in this example depicted, the elongation complex slips backward one base, landing on a template location where six of the nine bases in the RNA:DNA hybrid are not complementary. If transcription cannot resume due to the extent of mispairing in the RNA:DNA hybrid and/or fraying at the end of the transcript, the transcript is aborted (IV). Alternatively, if the elongation complex slips to template location with fewer mismatches, in this case a homopolymeric run (V), the 3’-end of the RNA bonds sufficiently to the DNA template, and transcription resumes (VI) after the re-transcribed region, generating an insertion.
